# Tailoring Biologic Therapies for Pediatric Severe Asthma: A Comprehensive Approach

**DOI:** 10.3390/children12020140

**Published:** 2025-01-27

**Authors:** Claudia María Chaverri Repáraz, Esther Lacalle Fabo, María Erroz Ferrer, María Gimeno-Castillo, Isabel Castro-Garrido, Miren Ibarzabal-Arregi, Nerea González Arza, Natividad Viguria, Laura Moreno-Galarraga

**Affiliations:** 1 Department of Pediatrics, Hospital Universitario de Navarra (HUN), 31008 Pamplona, Spain; 2 Department of Pharmacy, Hospital Universitario de Navarra (HUN), 31009 Pamplona, Spain; 3 Instituto de Investigación Sanitaria de Navarra (IdiSNA), 31008 Pamplona, Spain; 4 Medicina, Facultad de Ciencias de la Salud, Universidad Pública de Navarra (UPNA), 31006 Pamplona, Spain

**Keywords:** pediatric severe asthma, children, adolescents, biologic therapies, individualized management

## Abstract

**Introduction:** Biologic therapies have revolutionized the management of severe asthma in pediatrics, offering targeted options for specific inflammatory pathways. This study aimed to review the current indications and availability of biologics approved for pediatric use as of January 2025 and to analyze the clinical experience of a tertiary center in managing this condition. **Methods:** A comprehensive review of the biologics available for treating severe asthma in children was conducted, highlighting their indications and key characteristics. Additionally, a retrospective analysis was conducted on the experience of the Pediatric Severe Asthma Unit at the University Hospital of Navarra in utilizing these therapies between 2015 and 2025, with a focus on treatment distribution and reasons for switching biologics. **Results:** As of January 2025, the biologics available for pediatric use include omalizumab, mepolizumab, dupilumab, tezepelumab, and benralizumab, each with specific indications and different administration profiles. At the analyzed center, the distribution of biologics was as follows: omalizumab (27%), mepolizumab (27%), dupilumab (37%), and tezepelumab (9%). At the same center, the biologic distribution 10 years earlier was 100% omalizumab. Therapy changes and switches were primarily related to clinical response, posology, and comorbidities. **Conclusions:** This study emphasizes the importance of individualized management in pediatric severe asthma, based on continuous evaluation and appropriate biologic selection according to the clinical characteristics of each patient. It also highlights the need to develop specific guidelines for adjusting, switching, and discontinuing these therapies.

## 1. Introduction

Asthma is a chronic respiratory disease characterized by airway obstruction and hyperresponsiveness, with inflammation of the airways, resulting from exposure to allergens or other environmental irritants [1]. It is currently the most frequent chronic inflammatory disease in childhood [2]. Severe asthma affects 5–10% of children with asthma, and new biologics drugs are included in GINA step 5 and GEMA step 6 for the treatment of these patients [3,4].

The advent of biological therapies has revolutionized the management of severe asthma, offering targeted treatment options that address specific inflammatory pathways [5]. In pediatric populations, the availability of these biologics has been limited due to age restrictions; however, recent approvals have expanded their use to younger age groups, broadening the therapeutic arsenal for clinicians [6]. 

The most used biologics in pediatric asthma currently include:

- Omalizumab (Xolair^®^): An anti-IgE monoclonal antibody that binds to free IgE, preventing its attachment to mast cells and basophils, thereby inhibiting the allergic cascade [7,8]. It is indicated for children aged 6 years and older with moderate to severe persistent allergic asthma [9]. It is administered subcutaneously every 2 to 4 weeks, with the dosage determined by baseline IgE levels and body weight [10,11]. The use of this monoclonal antibody, the first one available in pediatric age groups, has proven to be a safe and cost-effective option for most children with uncontrolled severe allergic asthma [12] and is considered an effective and safe long-term treatment for children with severe asthma [13].
- Mepolizumab (Nucala^®^): An anti-IL-5 monoclonal antibody that inhibits eosinophilic inflammation by blocking interleukin-5. It is approved for children aged 6 years and older with severe eosinophilic asthma and is administered subcutaneously every 4 weeks at a fixed dose (not weight-dependent) [14].
- Dupilumab (Dupixent^®^): A monoclonal antibody targeting the IL-4 receptor alpha subunit, inhibiting IL-4 and IL-13 signaling pathways involved in type 2 inflammation. It is indicated for children aged 6 years and older with moderate to severe asthma with an eosinophilic phenotype or with oral corticosteroid-dependent asthma. It is administered subcutaneously every 2–4 weeks, with dosing based on weight and comorbidities [15].
- Tezepelumab (Tezspire^®^): A monoclonal antibody that targets thymic stromal lymphopoietin (TSLP), a key epithelial cytokine involved in the initiation of allergic inflammation. It is approved for adolescents aged 12 years and older with severe asthma, regardless of phenotype, and administered subcutaneously every 4 weeks at a fixed dose [16].
- Benralizumab (Fasenra^®^): An anti-IL-5 receptor alpha monoclonal antibody that induces direct, rapid, and near-complete depletion of eosinophils. It is indicated for adults (and adolescents aged 12 years and older in the USA) with severe eosinophilic asthma and is administered subcutaneously every 4 weeks for the first three doses, then every 8 weeks thereafter [17]. Selecting the appropriate biologic for each pediatric patient requires a comprehensive understanding of the pharmacological profiles, indications, and administration protocols of these therapies [18,19]. Tailoring treatment to the individual patient’s clinical characteristics and needs is essential for optimizing outcomes [20]. Today, with the wide variety of alternative treatment options available, it is critical not only to select the most appropriate biologic drug for each patient, whether they are naive to biologic therapies or already under treatment, but also to conduct ongoing reviews of children receiving these treatments [21]. Regular assessments allow for timely switches from one biologic to another if necessary, ensuring that the therapy remains as effective as possible while adapting to the evolving clinical needs of each patient [3,4].

## 2. Materials and Methods

This study provides a comprehensive review of the biologic therapies commonly used in pediatric patients with severe asthma, detailing their indications and administration protocols. Additionally, we present the clinical experience of the Severe Asthma Clinic (SAC) of a tertiary hospital center in managing such cases.

The study was performed in Navarra, located in northern Spain, a region with a population of 682,201, with 14.8% being children and adolescents [22,23]. The University Hospital of Navarra (Hospital Universitario de Navarra HUN) in Pamplona is the tertiary referral center for the region, managing approximately 100,000 children under 15 years of age, with an annual average of 44,000 pediatric emergencies, 2850 hospital admissions, and over 3400 births. The Pediatrics Department comprises more than 30 specialists, covering all subspecialties, and includes both NICU and PICU units. The Pediatric Pulmonology Department, with just two specialists, serves as the respiratory reference center for the region’s pediatric population. Annually, around 500 children are attended on first visits and over 2000 children on successive visits. It operates a specialized severe asthma clinic (HUN-SAC) and it has been accredited by the Spanish Society of Pulmonology and Thoracic Surgery (SEPAR). Patients followed in the SAC are monitored monthly with a clinical visit including pulmonary function tests and FeNO (fractional exhaled nitric oxide). Over the past decade, the therapeutic approach to severe asthma has shifted significantly: previously, only omalizumab was available in pediatric age, but different biologics have recently been introduced, broadening treatment options.

This paper aims to present the experience of our center in managing severe asthma, highlighting the therapeutic evolution and the clinical outcomes achieved over the past 10 years.

This study was conducted in accordance with the ethical standards set forth in the Declaration of Helsinki and complied with applicable local regulations concerning human subject research. Notably, the research focused on pharmacy prescription data rather than individual patient information. Nevertheless, all participants enrolled in our Severe Asthma Clinic have provided written informed consent. This informed consent is obtained from all patients and caregivers prior to initiating any treatment with biologics, and includes information about the specific biologic treatment, patient responsibilities regarding appointments and medication handling, authorization for the use of clinical data for research purposes, and acknowledgment of therapeutic alternatives, risks, and benefits.

## 3. Results

The first step in our study involved a comprehensive review and evaluation of the indications for biologic therapies in severe asthma, resulting in the creation of a detailed table (Table 1) that includes trade names, manufacturers, target molecules, indications, approved age groups, dosage, administration frequency, and other relevant information on the biological treatments available for pediatric severe asthma in Spain as of January 2025.

**Figure 1:**
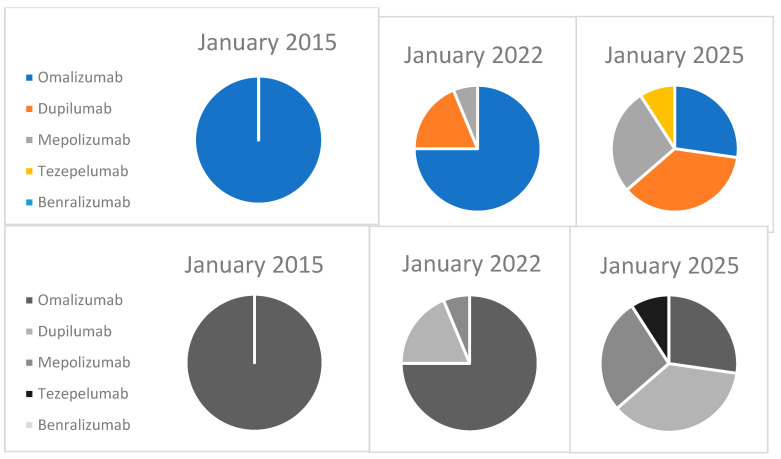
Evolution of biologic therapy utilization in children with severe asthma at SAC-HUN (2015–2025).

The second step in our study focused on describing the experience of our SAC in managing severe asthma, including the therapeutic approaches, patient monitoring protocols, and the evolution in the use of biologic therapies over the past decade. The evolving landscape of biologic therapy utilization in our Severe Asthma Clinic is illustrated in Figure 1. In 2015, all patients were treated with omalizumab. By 2020, approximately 20% of patients had transitioned to dupilumab, and as of January 2025, the distribution of biologic therapies is as follows: omalizumab: 27%, mepolizumab: 27%, dupilumab: 37%, and ezepelumab: 9%. Table 2 illustrates the evolution of biologic therapy use in pediatric patients with severe asthma over the past decade, including the number of patients treated, the duration of treatment for each biologic (in days), the introduction of new therapies, and the transitions between treatments.

This figure illustrates the progressive changes in biologic therapy use in the Severe Asthma Clinic at HUN over the past decade. In 2015, omalizumab was the only biologic used. By 2020, dupilumab was introduced, and in 2025, the distribution includes multiple biologics, reflecting the diversification of therapeutic options.

There are several reasons why a biologic therapy may be switched in the management of pediatric severe asthma. First, a change may be necessary if the child is not adequately controlled with the initial biologic, requiring a different therapy to achieve better asthma control. Second, side effects or intolerance to the current biologic might make it unsuitable for the patient, prompting a switch to a better-tolerated option. Third, comorbidities present in the child may be more effectively managed with an alternative biologic that addresses both asthma and the comorbid condition. Fourth, changes in the indications for existing biologics can lead to a reevaluation of the patient’s profile; a child whose characteristics now align with the updated indications for a biologic may benefit from a switch. Fifth, factors related to administration, such as reducing the number or frequency of injections, can also influence the decision. Finally, cost considerations play a role, as clinicians strive to select the most cost-effective biologic therapy that provides the same level of efficacy. In Spain, while specific clinical criteria guide the use of biologics, there are also local- and hospital-specific guidelines and criteria defining which drugs are covered by the Spanish national healthcare system, in addition to the physician’s clinical judgment. Physicians generally have the freedom to prescribe the medication they consider most appropriate.

In our center’s experience, switches to dupilumab were primarily made for children with poorly controlled atopic dermatitis, as these biologic addresses both severe asthma and this comorbidity. The high number of patients who switched to dupilumab is due to the large proportion of patients with severe asthma who also had atopic dermatitis as a comorbidity. At that time, dermatologists in our center were not yet using dupilumab in children, so many of our patients with severe asthma and atopic dermatitis switched primarily due to skin-related issues. The switch to tezepelumab was motivated by its dosing schedule, which significantly reduced the number of injections per year (the number of injections decreased from over 90 shots/year to 12 shots/year and the frequency of visits decreased from every two weeks to once a month, improving adherence to treatment, and overall patient satisfaction). Additionally, the switch maintained good clinical control and even resulted in improved pulmonary function.

This diversification reflects the expanding therapeutic options and the tailored approach adopted for each patient based on continuous clinical evaluation.

## 4. Discussion

A thorough understanding of biologic therapies is crucial for aligning the most suitable treatment with each pediatric patient suffering from severe asthma. This alignment should be assessed at the initiation of therapy and continually re-evaluated over time. Reasons for switching biologics may include suboptimal response to the initial therapy, the presence of comorbidities better addressed by an alternative biologic, more convenient dosing schedules, cost considerations, or other advantages.

The paradigm of asthma management has evolved significantly with the advent of biologic therapies. These are not a substitute for inhaled treatment. Despite the good effectiveness of biological treatments, it is essential to focus on other critical aspects of managing severe asthma in children, including:

- **Education of the child and parents:** Learning to recognize the symptoms of asthma exacerbation and their appropriate treatment; proper inhalation techniques for using inhalers and nebulizers; avoiding triggers (e.g., allergens, tobacco smoke, infections, cold air) and maintaining good adherence to basal treatments. Therapeutic education is undoubtedly the cornerstone of asthma treatment.
- **Prevention of respiratory infections:** Vaccinations against influenza and pneumococcal disease.
- **Avoiding tobacco smoke:** Reducing exposure to passive smoking/vaping.
- **Physical activity:** Encouraging moderate physical activity to improve respiratory efficiency or respiratory rehabilitation if needed.
- **Psychosocial support:** Providing psychological support to help children and their families cope with chronic disease and collaborating with schools and teachers to ensure a safe environment.

The integration of all these aspects with biological treatments is essential to achieving optimal asthma control.

Continuous monitoring and reassessment of patients are imperative, as both patient characteristics and therapeutic indications may change over time. Ensuring the appropriateness of treatment is essential for optimal patient outcomes. Our findings align with current observations that the choice of biologic therapy is influenced by a combination of factors, including age, company-defined criteria, comorbidities, cost, frequency of administration, and reimbursement policies. Furthermore, we acknowledge the need for more data to establish clear guidelines on patient profiles for each biologic, optimal duration of treatment, and criteria for discontinuation. Guidelines for the discontinuation of biologic therapies in pediatric patients remain underdeveloped. While initiation protocols and indications are well-defined, there is a lack of consensus on the timing and method of withdrawal. Multidisciplinary studies are needed to assist pediatricians in making informed decisions regarding the cessation of biologic treatments, including whether to taper doses gradually or discontinue abruptly and the appropriate timing for such actions.

There is a need for further studies and multicenter collaborations, as well as consensus from relevant societies, regarding the switch between different biologics and the strategies for discontinuing these treatments. In our center, SAC, biologic therapy typically lasts between 4 to 6 years. After this period, we begin a gradual withdrawal process: for treatments administered every two weeks, the intervals are progressively extended, and for monthly treatments, the dose is initially reduced. If clinical response and control remain stable after six months of gradual tapering, discontinuation is considered. These patients are followed for several months in the SAC. Based on our experience, outcomes are generally favorable, and patients are either referred to adult pulmonology or the regular pediatric asthma clinic. However, there are no specific protocols on when or how to stop biologics. Multicenter studies and working groups are essential to establish not only the initiation (which is already well-defined in terms of dosage and monitoring) but also the optimal approach for follow-up and discontinuation.

## 5. Conclusions

In conclusion, with the growing availability of new drugs, we must continuously review comorbidities, clinical responses in children, and the evolving indications for these therapies, which are continuously changing as the age for initiating biologic treatment decreases. By doing so, we can ensure that each child receives the most appropriate drug, tailoring treatment to each patient in an individualized manner. While biologics have shown significant effectiveness in managing severe asthma, it is essential to consider patient-specific factors, including clinical response, adverse effects, and individual needs, to optimize outcomes. Further research is necessary to establish standardized criteria for switching and discontinuing treatment.

## Figures and Tables

**Table 1:** Overview of biologics for severe asthma in pediatric populations (Spain, January 2025).

	Omalizumab	Mepolizumab	Dupilumab	Tezepelumab	Benralizumab
**Trade Name**	Xolair^®^	Nucala^®^	Dupixent^®^	Tezspire^®^	Fasenra^®^
**Presentation**	75–150 mg syringes	40 mg syringe and 100 syringe and pen	200–300 mg syringe and pen	210 mg syringe and pen	30 mg syringe and pen
**Manufacturer**	Novartis (Basel, Switzerland)	GlaxoSmithKline (Brentford, UK)	Sanofi (Paris, France)	AstraZeneca (Cambridge, UK)	AstraZeneca (Cambridge, UK)
**Target Molecule**	IgE	IL-5	IL-4Rα, (IL-4 and -13)	TSLP	IL-5Rα
**Indication in Asthma**	Moderate to severe uncontrolled allergic asthma in patients ≥ 6 years Indicated as add-on therapy for patients ≥ 6 years old with severe allergic asthma, positive skin test or iv reactivity perennial aeroallergens, reduced lung function (FEV1 < 80%) (≥12 years), frequent symptoms or night awakenings, and severe exacerbations despite high-dose ICS-LABA	Severe eosinophilic asthma in patients ≥ 6 years old Indicated as an add-on treatment for severe refractory eosinophilic asthma patients ≥ 6 years old	Moderate to severe asthma with type 2 inflammation in patients ≥ 6 years old Indicated in patients ≥ 6 years old as add-on treatment for severe asthma with type 2 inflammation characterized by raised blood eosinophils and/or raised fraction of exhaled nitric oxide (FeNO), inadequately controlled with medium- to high-dose ICS plus another maintenance treatment	Severe uncontrolled asthma in patients ≥ 12 years old, regardless of phenotype Indicated as an add-on maintenance treatment in adults and adolescents 12 years and older with severe asthma who are inadequately controlled despite high-dose ICS plus another maintenance treatment	Currently indicated as add-on therapy for adults (adolescents ≥ 12 years old in the USA) with severe eosinophilic uncontrolled asthma despite high-dose ICS plus another maintenance treatment Future indications may soon expand to younger patients
**Funding Restrictions in Spain (Spanish Public Healthcare System)**	Unrestricted	Funding is restricted to patients with: - Eo ≥ 500/µL - Eo < 500/µL but with more than 2 severe exacerbations in the past year requiring the use of ≥2 cycles GC or an increase in maintenance dose for >3 days, or ≥1 severe exacerbation requiring hospitalization, ICU admission, or mechanical ventilation	Funding is restricted to patients with: - Eo ≥ 300 µL - FeNO ≥ 50 EoS 150–300 µL but with more than 2 severe exacerbations in the past year or more than 1 requiring hospitalization, or with chronic use of oral corticosteroids	Funding is restricted to patients with: - ≥2 exacerbations requiring treatment with oral or systemic corticosteroids - ≥1 hospitalization for exacerbation in the previous year.	Funding is restricted to patients with: - severe refractory eosinophilic asthma Eo ≥ 500/µL - Eo < 500 µL but with >2 exacerbations in the past year requiring use of 2 cycles of corticosteroids or increased dose of corticosteroids for 3 days, or ≥1 exacerbation requiring hospitalization, ICU admission, or mechanical ventilation
**Other Indications**	CSU (≥12 years) CRwNP (adults)	EGPA (≥6 years) HES (adults) CRSwNP (adults)	AD (≥6 months) CRSwNP (adults) PN (adults) EoE (≥1 year) COPD (adults)	Currently indicated only for asthma	Currently indicated only for asthma
**Dosage in Asthma**	Subcutaneous; dose based on weight and IgE levels 75 to 600 mg in 1 to 4 injections	Subcutaneous; 100 mg or 40 mg depending on age 6–11 years: 40 mg ≥12 years: 100 mg	Subcutaneous; dose based on body weight and co-morbidities	Subcutaneous; fixed dose of 210 mg	Subcutaneous; fixed dose of 30 mg
**Frequency of Administration (asthma)**	Every 2 or 4 weeks	Every 4 weeks	Every 2 or 4 weeks	Every 4 weeks	Every 4 weeks for the first 3 doses, then every 8 weeks
**Cost Considerations**	High cost; varies by dosing, local agreements, and country	High cost; price may vary depending on country and local agreements	High cost; price may vary depending on country and local agreements	High cost; price may vary depending on country and local agreements	High cost; price may vary depending on country and local agreements

This table summarizes the main characteristics of biologics used in the management of severe asthma, including trade names, manufacturers, target molecules, indications, approved age groups, dosage, administration frequency, cost considerations, and public funding restrictions in Spain. Additional indications beyond asthma are also noted. Abbreviations used in the table: Eo: Eosinophils (measured in cells/µL), CSU: Chronic Spontaneous Urticaria, CRSwNP: Chronic Rhinosinusitis with Nasal Polyps, EGPA: Eosinophilic Granulomatosis with Polyangiitis, HES: Hypereosinophilic Syndrome, AD: Atopic Dermatitis, EoE: Eosinophilic Esophagitis, COPD: Chronic Obstructive Pulmonary Disease, FeNO: Fraction of Exhaled Nitric Oxide, ICS: Inhaled Corticosteroids, LABA: Long-Acting Beta-Agonist, GC: Glucocorticoids, ICU: Intensive Care Unit.

**Table 2:** SAC-HUN patient distribution, treatment duration, and transitions in biologic therapies for pediatric severe asthma over the past decade (2014–2025) This table illustrates the evolution of biologic therapy use in pediatric patients with severe asthma over the past decade, including the number of patients treated, the duration of treatment for each biologic, the introduction of new therapies, and the transitions between treatments.

Pacients	2014	2015	2016	2017	2018	2019	2020	2021	2022	2023	2024	2025	Persistency (In Days)
1													998
2													98
3													823
4													1390
5													1901
6													1112
7													1013
8													557
9													14
10													1309
11													1246
12													1995
13													337
14													938
15													1149
16													1806
17													2618
18													1085
19													1491
20													1516
21													2002
22													1883
23													2156
24													1575_371
25													1113_924
26													998_874
27													1764
28													756_531
29													231_651
30													1050_203
31													1148
32													882
33													636
34													322
35													301

Note: 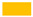 OMALIZUMAB; 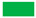 DUPILUMAB; 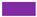 MEPOLIZUMAB; 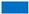 TEZEPELUMAB.

## Data Availability

No new data were created for this article. The study involved a review of medications used over the last 10 years and did not include data creation or statistical analysis.
